# New Insights into the Molecular Actions of Grosheimin, Costunolide, and α- and β-Cyclocostunolide on Primary Cilia Structure and Hedgehog Signaling

**DOI:** 10.3390/ijms262311754

**Published:** 2025-12-04

**Authors:** Marina Murillo-Pineda, Joel Martínez-Miralles, Zahara Medina-Calzada, Rosa María Varela, Francisco Antonio Macías, Nuria Chinchilla, Álvaro Juárez-Soto, Gabriel Santpere, Elena Reales

**Affiliations:** 1 Urology Clinical Unit, Biomedical Research Institute of Cadiz (INiBICA), University Hospital of Jerez, 11407 Jerez, Spain; marina.murillo@inibica.es (M.M.-P.); zahara.medina@inibica.es (Z.M.-C.); alvaro.juarez.sspa@juntadeandalucia.es (Á.J.-S.); 2 Neurogenomics Group, Hospital del Mar Research Institute, Parc de Recerca Biomèdica de Barcelona (PRBB), Dr. Aiguader, 88, Catalonia, 08003 Barcelona, Spain; marmirjoel@gmail.com (J.M.-M.); gabrielsantperebaro@gmail.com (G.S.); 3 Department of Organic Chemistry, Faculty of Sciences, Institute of Biomolecules (INBIO), University of Cadiz, 11510 Puerto Real, Spain; rosa.varela@uca.es (R.M.V.); fmacias@uca.es (F.A.M.); nuria.chinchilla@uca.es (N.C.)

**Keywords:** sesquiterpene lactones, primary cilium, Hedgehog pathway, ciliopathies, tumor, grosheimin, costunolide

## Abstract

Sesquiterpene lactones (SLs) are plant-derived metabolites with recognized pharmacological properties. Dysfunction of the primary cilium (PC), a solitary sensory organelle essential for development, is associated with disorders such as ciliopathies and tumors. While previous studies have shown that certain SLs can alter PC structure in human retinal cells, their influence on ciliary signaling pathways remains unclear. In this study, we examined the effect of four SLs—grosheimin, costunolide, α-cyclocostunolide (α-C), and β-cyclocostunolide (β-C)—on ciliary function in human primary fibroblasts. Using immunofluorescence and qPCR to assess cilia structure and Hedgehog (Hh) pathway activation, we found that grosheimin enhanced ciliogenesis without affecting Hh signaling. In contrast, costunolide, α-C, and β-C disrupted ciliary structure and suppressed the Hh pathway transcripts *Gli1* and *Ptch1.* RNA sequencing revealed that grosheimin upregulated genes related to microtubule binding and ciliogenesis, whereas α-C downregulated tubulin subunit transcripts. These findings suggest distinct molecular mechanisms through which SLs affect ciliary structure and function. Collectively, this study highlights the potential of specific SLs as modulators of ciliary signaling, offering promising leads for therapeutic strategies targeting ciliopathies and tumors.

## 1. Introduction

Primary cilia (PC) are solitary sensory organelles that extend from the cell surface in nearly all cell types. These structures function as signaling hubs that integrate extracellular cues essential for embryonic development, tissue homeostasis, and cellular communication [1]. They consist of a microtubule-based axoneme surrounded by a specialized ciliary membrane enriched in receptors and effector molecules and are nucleated from the older of the two centrioles, known as the basal body. Cilia are dynamic structures whose formation (ciliogenesis) occurs predominantly in non-dividing or post-mitotic differentiated cells, and they are disassembled prior to mitosis. These cellular mechanisms are tightly regulated through vesicular trafficking, cytoskeletal remodeling, and membrane fusion events [1,2]. Once formed, cilia maintain a specific length in each cell type, which is vital for their signaling function [3,4].

Primary cilia play key roles in the regulation of multiple signaling pathways involved in development, including Hedgehog (Hh), Wingless/Int (Wnt), and Transforming Growth Factor-b (TGF-β) pathways [1,5]. In the canonical Hh pathway, which is strictly dependent on the primary cilium, activation begins with the binding of Sonic Hedgehog (Shh) ligands to the Patched1 (PTCH1) ciliary receptor, relieving its repressive effect on Smoothened (SMO), a G-protein-coupled receptor. This allows SMO to accumulate in the cilium, triggering the activation of GLI transcription factors, which subsequently translocate to the nucleus to drive expression of Hh target genes such as *Gli1* and *Ptch1* [6,7]. Defects in the integrity or function of the primary cilium are associated with a heterogeneous group of human developmental disorders collectively referred to as ciliopathies [8,9]. Ciliopathies comprise more than 30 human diseases caused by pathogenic variants in ciliary genes. Since cilia are present in most cell types, these diseases affect nearly every major organ, including the brain, eye, and kidney, highlighting the critical role of PC in tissue homeostasis and development. Moreover, the presence or absence of PCs has been associated with different types of tumors, influencing cancer progression and therapy resistance [10,11]. Interestingly, recent studies have shown that current anticancer drugs can alter ciliation, with unexpected consequences for disease progression [12].

Plants are an important source of natural compounds with a wide spectrum of pharmacological activities, particularly in oncology [13]. Among these, sesquiterpene lactones (SLs)—a large class of plant-derived secondary metabolites—have shown promising roles in drug development [14]. Their biological activities are often associated with the inhibition of key enzymes such as farnesyltransferase or modulation of pathways like Ras, NF-kB, and apoptosis-related mechanisms [14,15,16,17]. However, their role in modulating cilia structure and cilia-mediated signaling pathways remains poorly understood. Our previous work identified a novel activity of selected SLs on PC formation and elongation in retinal pigment epithelial (RPE) cells—a non-tumoral cell line commonly used to study ciliogenesis in vitro—at non-toxic doses and without altering the cell cycle [18]. Among them, grosheimin, an SL isolated from *Cynara scolymus* L. (artichoke) leaves, enhanced PC formation and elongation under late ciliogenesis conditions. In contrast, costunolide, extracted from the roots of *Saussurea costus*, and three chemically synthesized derivatives—α-cyclocostunolide (α-C), β-cyclocostunolide (β-C), and γ-cyclocostunolide (γ-C) [19]—disrupted PC formation irrespective of cell cycle or actin cytoskeleton perturbations.

Based on these findings, we hypothesized that specific SLs could modulate ciliary signaling. Here, we investigate the effects of grosheimin, costunolide, α-C, and β-C on primary cilium structure and Hedgehog (Hh) pathway activity in human primary fibroblasts. Our results indicate that grosheimin enhances cilium formation and modestly increases Hh target gene expression, whereas costunolide, a-C, and b-C disrupt ciliary architecture and impair Hh signaling. These observations suggest that selected SLs can modulate cilia-associated pathways, providing a rationale for their potential therapeutic application in ciliopathies and cilia-dependent tumors

## 2. Results

### 2.1. Sesquiterpene Lactones Modulate Primary Cilium Formation in Human Fibroblasts

**Figure 1:**
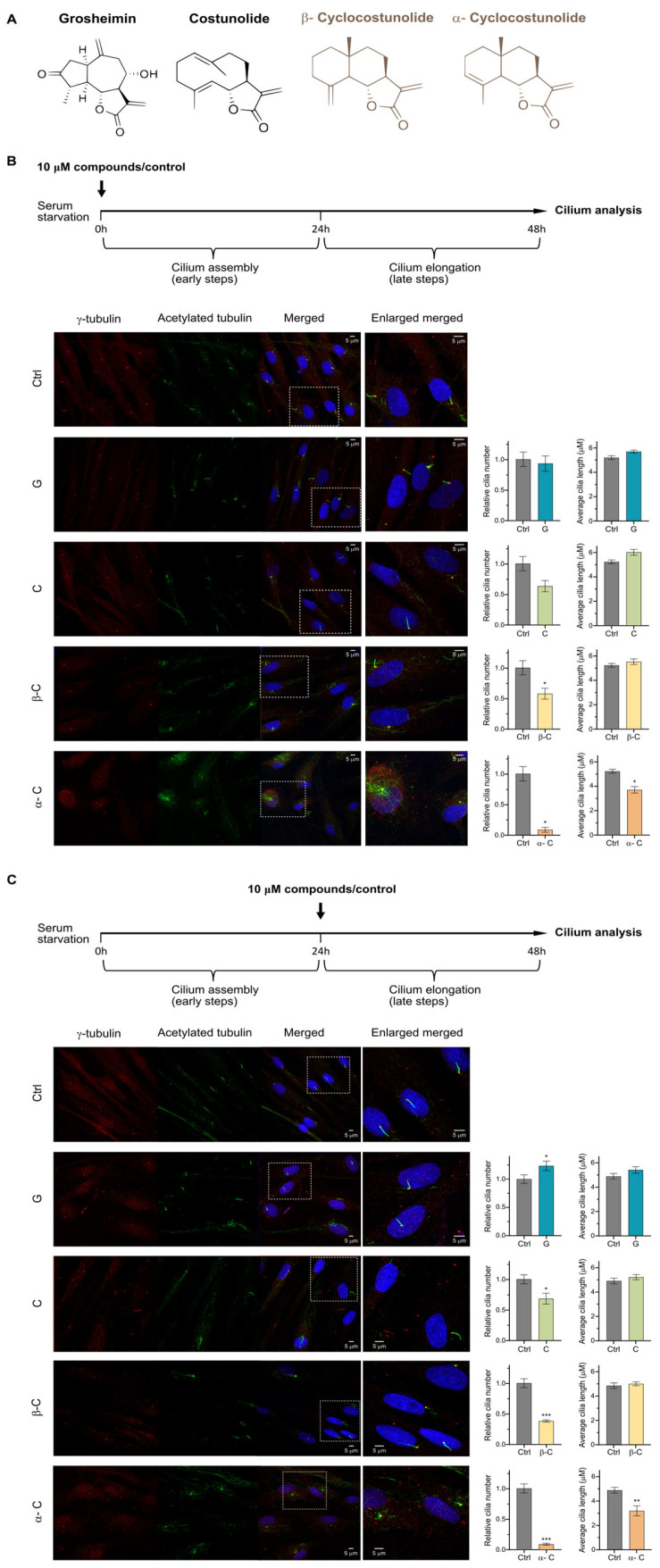
Effect of sesquiterpene lactones on primary cilium formation and assembly in fibroblasts. (**A**) Chemical structures of the sesquiterpene lactones (SLs) used in this study. (**B**) Cilia formation assay. Fibroblasts were incubated for 24 h in medium containing 0.5% serum to induce ciliogenesis, and compounds were added simultaneously at a final concentration of 10 μM. (**C**) Cilia elongation assay. Fibroblasts were first incubated for 24 h in 0.5% serum medium to induce primary cilia formation, and then treated with compounds at a final concentration of 10 μM for an additional 24 h in the same medium. (**B**,**C**) Ctrl: 0.1%DMSO. Immunofluorescence staining was performed using acetylated α-tubulin to label primary cilia and γ-tubulin to visualize centrosomes. Representative images for each condition are shown. Graph shows mean and **±** SEM from four (cilia formation) and five (cilia elongation) independent experiments, representing the percentage of ciliated cells and the average cilia length. Mann–Whitney test analysis was performed, and significant differences are indicated as *p* < 0.05 (*), 0.01 (**), 0.001 (***). SL labels: Grosheimin (G); Costunolide (C); α-Cyclocostunolide (α-C); and β-Cyclocostunolide (β-C).

First, we sought to determine whether the changes in primary cilium (PC) formation and stability previously observed in SL-treated RPE cells [18] were evident in a different primary cell type, such as fibroblasts. Human fibroblasts isolated from skin were treated with 10 μM of grosheimin, costunolide, α-cyclocostunolide (α-C), and β-cyclocostunolide (β-C)—a non-cytotoxic concentration—under serum-reduction conditions to induce cilia assembly (early ciliogenesis) and elongation (late ciliogenesis) (Figure 1A). Cilia number and length were quantified and analyzed by immunofluorescence microscopy using acetylated-tubulin and γ-tubulin as markers for axoneme and centrosome, respectively (Figure 1).

We found that costunolide, α-C, and β-C reduced the percentage of ciliated cells under both early ciliogenesis and late ciliogenesis treatment conditions, as shown in Figure 1B and Figure 1C, respectively. In contrast, grosheimin increased the percentage of ciliated cells during late ciliogenesis (cilium stability) phase, while showing no significant or only a mild increase under early ciliogenesis conditions (Figure 1B,C). No major changes in cilium length were detected for any compound, except for α-C, which caused a notable shortening of the axoneme in both treatment conditions (Figure 1B,C). These results indicate that SLs differentially regulate primary cilium formation and stability in human fibroblasts. Altogether, the stimulatory effect of grosheimin and the inhibitory effects of costunolide and its derivatives are robust and consistent with our previous findings in RPE cells [18], suggesting that these responses are conserved across tissue-specific and non-polarized cell types. These data encouraged us to further investigate the effect of SLs on signaling pathways dependent on the PC.

### 2.2. Sesquiterpene Lactones Differentially Impair Hedgehog Signaling

Hedgehog (Hh) pathway intensity is directly influenced by the architecture of the PC, and failure in both cilium formation and trafficking of ciliary receptors have been associated with reduced Hh signaling [20,21]. Interestingly, Hh signaling has also been reported in non-ciliated immune cells [22], suggesting a cilia-independent mechanism that may explain why, in certain cell types, a reduction in PC does not always impair Hh activity [23,24]. To better understand the impact of SLs on PC-associated processes, we next analyzed whether SL-induced structural changes observed in cilia correlated with functional alterations, focusing specifically on the Hh signaling pathway.

**Figure 2:**
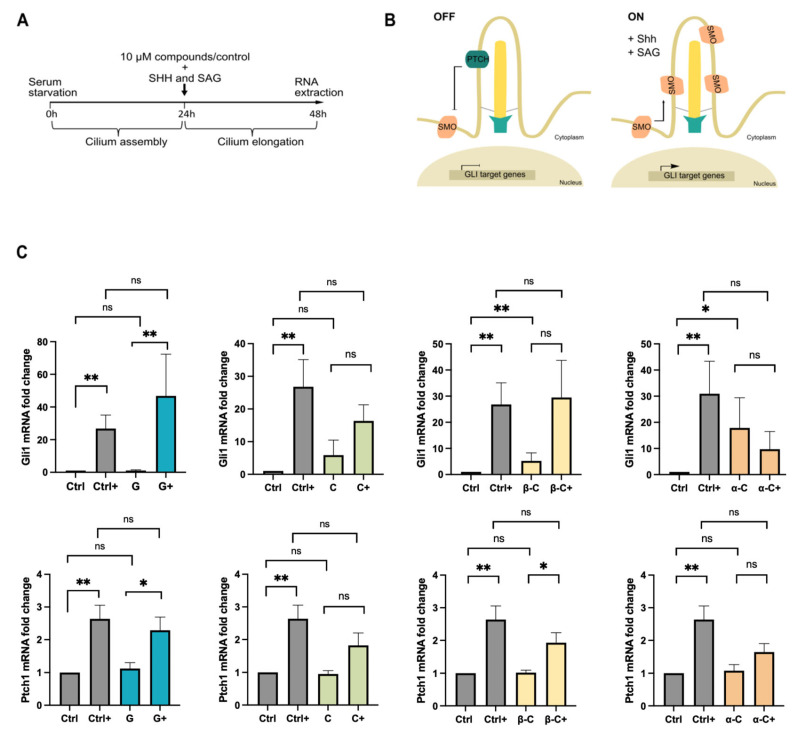
Effect of SLs on Hedgehog signaling. (**A**) Experimental timeline of Hh pathway activation. Compounds and Hh activators were added to the cells after 24 h of serum starvation and then incubated for an additional 24 h before RNA extraction. (**B**) Schematic representation of Hh pathway activation showing SMO/PTCH1 trafficking in the presence (ON) and absence (OFF) of Hh ligands. Activation (→) and repression (⌐) of Gli1 target gene transcription are indicated. (**C**) Relative *Gli1* and *Ptch1* mRNA levels in control (Ctrl) cells and SL-treated cells under Hh activation (+) or no-treatment (−). Data represent six biological replicates. Mann–Whitney test analysis was performed, and significant differences are indicated when *p* < 0.05 (*), 0.01 (**). SLs label: Grosheimin (G); Costunolide (C); α-Cyclocostunolide (α-C); and β-Cyclocostunolide (β-C).

Fibroblasts, a broadly ciliated cellular model, are able to activate the Hh pathway in response to stimulation with recombinant Sonic Hedgehog (Shh) ligand or Smoothened Agonist (SAG), leading to increased transcription of *Gli1* and *Ptch1* [25,26] (Figure 2B). Because Shh and SAG bind to different receptors—PTCH1 and SMO, respectively—they do not compete with each other and can be combined for full pathway activation. Primary human fibroblasts were serum-starved for 24 h to induce ciliogenesis, followed by an additional 24 h of starvation during which cells were treated with 10 μM of each SL and simultaneously stimulated with 750 nM of SAG and 60 ng/mL Shh-ligand for Hh pathway activation (Figure 2A). Expression levels of the canonical Hh target genes *Gli1* and *Ptch1* were then quantified by qPCR.

As shown in Figure 2C, stimulation with Shh and SAG induced a strong upregulation of *Gli1* and *Ptch1* transcripts in control cells (no SL treatment). Grosheimin-treated cells retained this response, showing similar or even slightly higher expression levels of both genes compared with untreated cells. These results indicate that grosheimin preserves Hh pathway responsiveness, suggesting that this biomolecule does not interfere with receptor trafficking to and from the cilium. This observation is consistent with its positive effect on primary cilium formation in both RPE and fibroblast cells.

In contrast, cells treated with costunolide, α- C, and β-C failed to upregulate *Gli1* and *Ptch1* expression upon Hh activation, except for β-C-treated cells, which showed increased *Ptch1* mRNA levels. This suggests an impairment of Hh signaling likely related to their structural effects on cilia. Interestingly, basal (unstimulated) *Gli1* and *Ptch1* levels were modestly elevated in α-C- and β-C-treated cells, indicating possible pathway deregulation independent of canonical Hh activation.

Collectively, these data demonstrate that SLs not only modify cilia structure but also cilia-dependent pathways such as Hedgehog. While grosheimin enhances or maintains ciliary Hh signaling, costunolide and its derivatives compromise the pathway, most likely by disrupting ciliogenesis or ciliary trafficking.

### 2.3. Transcriptomic Analysis of Grosheimin and a-Cyclocostunolide

To investigate the molecular mechanisms underlying the effects observed in SLs-treated cells, we performed RNA sequencing (RNA-seq) on untreated cells (DMSOs) and RPE cells treated with 10 μM grosheimin or 10 μM α-C under conditions promoting cilium elongation and maintenance (Figure 1C). These two biomolecules were selected based on their opposing effects on primary cilium formation and function, either enhancing or reducing it, respectively (Figure 1 and Figure 2).

**Figure 3:**
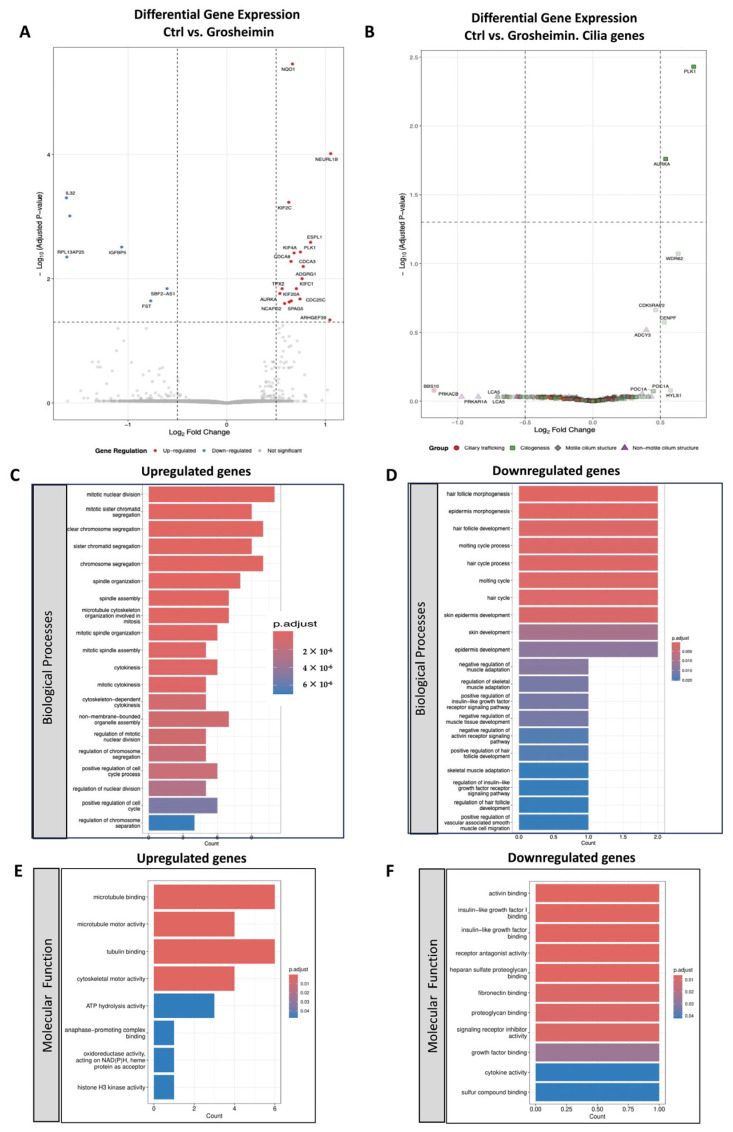
Differential expression genes in untreated cells versus grosheimin-treated cells. (**A**) Volcano plot showing log_2_ fold change versus −log_10_ (*p*-value) for all transcripts, and (**B**) for cilia-related genes referenced from [29], identified by RNA-seq analysis of untreated cells and grosheimin-treated cells. Genes with a ≥1.5-fold change and *p* < 0.05 were considered differentially expressed (23 genes). In (**A**), each dot represents a gene: red dots indicate significantly upregulated genes, blue dots indicate significantly downregulated genes, and gray dots indicate genes without statistically significant expression changes. In (**B**), red dots represent genes related to ciliary trafficking, green squares indicate genes involved in ciliogenesis, gray diamonds represent genes associated with motile cilium structure, and purple triangles denote genes associated with non-motile cilium structure. (**C**–**F**) Gene Ontology (GO) term enrichment analysis for biological processes and molecular function categories of the up- and downregulated genes. Significantly enriched GO terms are shown as bar plots. The *X*-axis represents the number of counts (gene products associated with each GO term) and the *Y*-axis represents the GO terms. The *p*-value of each term is color-coded from blue to red, indicating decreasing significance. GO Biological Process terms for upregulated (**C**) and downregulated (**D**) genes, and GO Molecular Function terms for upregulated (**E**) and downregulated (**F**) genes.

**Figure 4:**
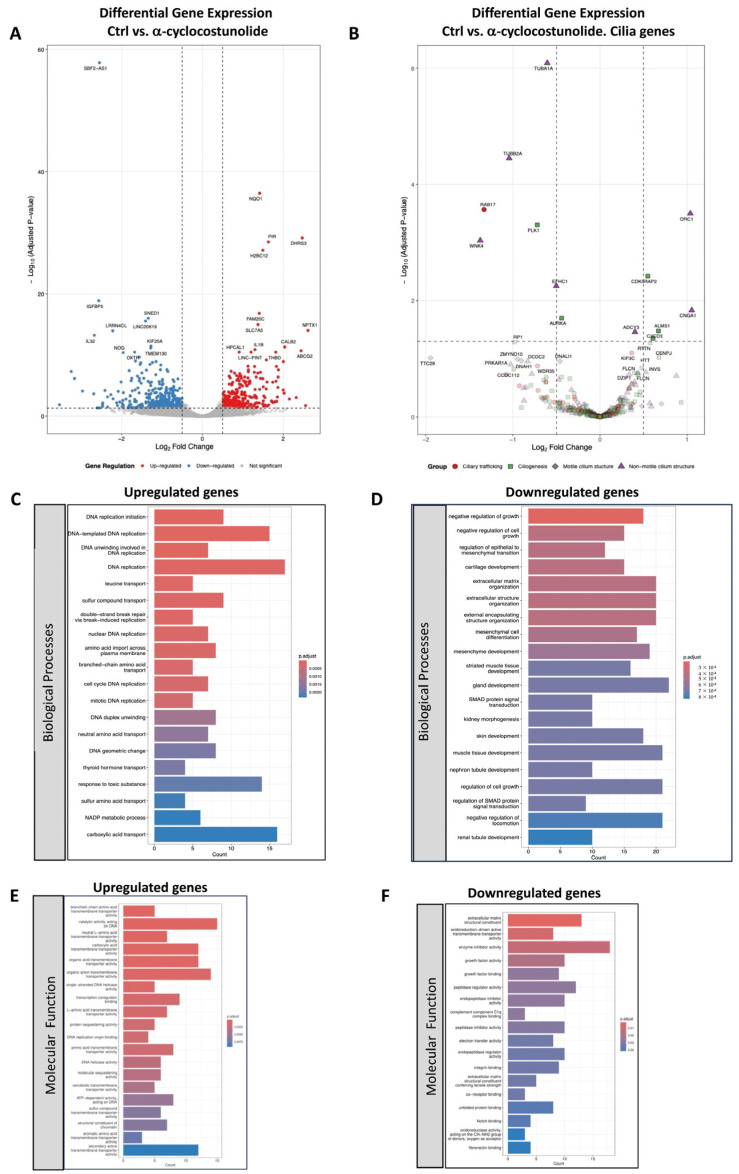
Differential expression genes of untreated cells versus a-cyclocostunolide-treated cells. (**A**) Volcano plot showing log_2_ fold change versus −log_10_ (*p*-value) for all transcripts, and (**B**) for cilia-related genes referenced from [29], identified by RNA-seq analysis of untreated cells and α-cyclocostunolide-treated cells. Genes with a ≥1.5-fold change and *p* < 0.05 were considered differentially expressed (725 genes). In (**A**), each dot represents a gene: red dots indicate significantly upregulated genes, blue dots indicate significantly downregulated genes, and gray dots indicate genes without statistically significant expression changes. In (**B**), red dots represent genes related to ciliary trafficking, green squares indicate genes involved in ciliogenesis, gray diamonds represent genes associated with motile cilium structure, and purple triangles denote genes associated with non-motile cilium structure. (**C**–**F**) Gene Ontology (GO) term enrichment analysis for biological processes and molecular function categories of the up- and downregulated genes. Significantly enriched GO terms are shown as bar plots. The *X*-axis represents the number of counts (gene products associated with each GO term) and the *Y*-axis represents the GO terms. The *p*-value of each term is color-coded from blue to red, indicating decreasing significance. GO Biological Process terms for upregulated (**C**) and downregulated (**D**) genes, and GO Molecular Function terms for upregulated (**E**) and downregulated (**F**) genes.

Differential expression analysis identified 23 genes (adjusted *p* < 0.05) as significantly regulated between control cells and grosheimin-treated cells, whereas 725 genes (*p* < 0.05) were differentially expressed in cells treated with α-C-treated cells. Volcano plots were generated to visualize differentially expressed genes (DEGs), as shown in Figure 3A for grosheimin and Figure 4A for α-C. Among these, the gene encoding NQO1 (*NAD(P)H:quinone oxidoreductase 1*), an antioxidant enzyme that protects cells against oxidative stress [27], was identified as the most significantly upregulated gene in both treatments, consistent with the antioxidant activity previously reported for certain SLs [28].

To determine the biological significance of differentially expressed genes, Gene Ontology (GO) enrichment analysis was performed. Significantly GO terms were identified within the biological process and molecular function categories. Grosheimin treatment led to the overexpression of genes involved in microtubule organization, mitotic spindle assembly, chromosome segregation, and cilium-associated structures, as shown by the over-representation analysis (ORA) in Figure 3C (GO Biological Process). Molecular function analysis of upregulated DEGs under grosheimin treatment showed enrichment in microtubule binding, microtubule motor activity, and tubulin binding (Figure 3E). Downregulated genes under grosheimin treatment were relatively few and were mainly associated with morphogenesis (Figure 3D,F).

In contrast, α-C treatment resulted in the downregulation of genes related to biological processes such as negative regulation of cell growth, regulation of epithelial-to-mesenchymal transition, and extracellular matrix organization (Figure 4D). Downregulated genes in the molecular function category were mainly associated with the collagen-containing extracellular matrix and endoplasmic reticulum lumen (Figure 4F). Conversely, GO terms for biological processes and molecular functions upregulated under α-C treatment included genes involved in DNA replication and molecular transport, as shown in Figure 4C and Figure 4E, respectively.

A more focused analysis of genes involved in ciliary processes showed that genes encoding kinases associated with the maturation of the centrosomal microtubule-organizing center (MTOC) [30], such as PLK1 and AURKA, as well as WDR62—an AURKA adaptor—and the centrosomal protein CENPF, all of which have known roles in ciliogenesis, were significantly upregulated following grosheimin treatment (Figure 3B). In contrast, α-C treatment led to a significant downregulation of genes encoding for tubulin subunits, including TUBA1A and TUBB2A, as well as PLK1, AURKA, and WNK4, while genes for ORC1 and ALMS1 were upregulated (Figure 4B).

**Figure 5:**
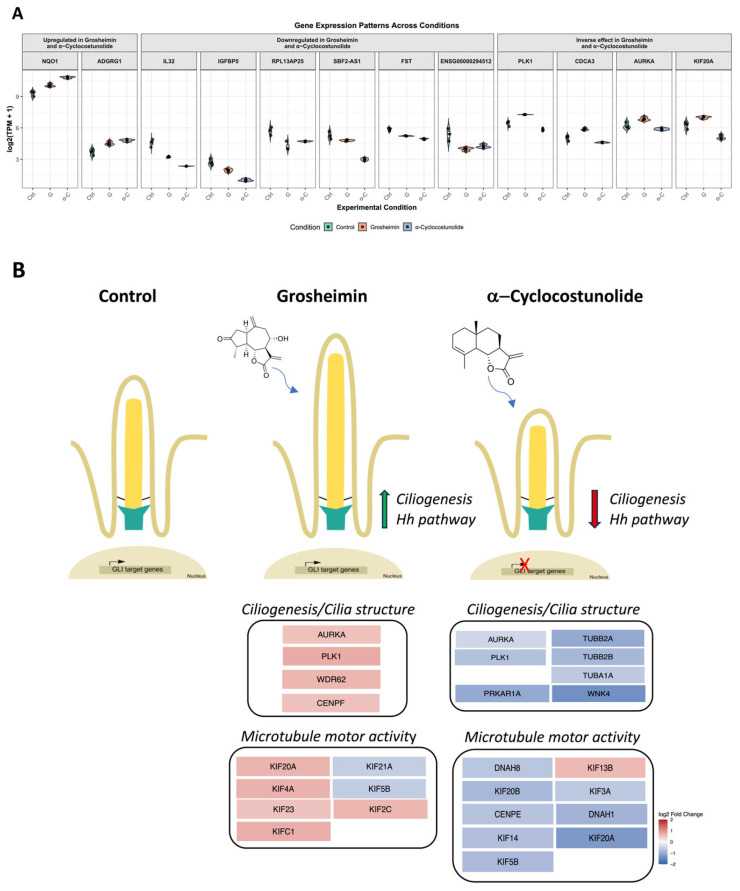
Proposed model of action for grosheimin and costunolide derivatives as modulators of primary cilium formation and function. (**A**) Comparative gene expression patterns across treatment conditions: control, groheimine, and α-cyclocostunolide treatments. *X*-axis: log_2_ (TPM + 1) expression values, where TPM (Transcripts Per Million) is transformed to stabilize variance and enable visualization of both low- and high-expression genes (from the RNA-seq data). (**B**) Schematic model summarizing the impact of SLs on primary cilium formation and ciliogenesis. Grosheimin enhances ciliogenesis and Hh pathway activity (green arrow), whereas α-cyclocostunolide impairs ciliogenesis and Hh pathway activity (red arrow). Differential gene expression under grosheimine and α-cyclocostunolide treatment of selected genes involved in ciliogenesis/cilia structure (referenced from [29]), and genes associated with microtubule motor activity function (GO:0003777), highlights the impact of microtubule stabilization or disruption as potential mechanisms underlying the observed effects. Color code indicating Log_2_ fold change for cilia transcripts.

Comparative gene expression analysis of grosheimin and α-C treatments revealed that both compounds upregulated *NQO1* and *ADGRG1A* (adhesion G protein-coupled receptor G1). Conversely, both SL treatments suppressed the expression of *IL32, IGFBP5, RPL13AP25, SBF2-AS1,* and *FST,* and displayed inverse regulation of *PLK1*, *CDCA3, AURKA,* and *KIF20A* (Figure 5A). Collectively, the transcriptome data support distinct molecular outcomes for the two SLs: grosheimin promotes the expression of microtubule- and spindle-related genes that favor cilium stability, whereas α-C represses structural tubulin genes, consistent with its inhibitory effect on ciliogenesis. Both compounds activate antioxidant responses, consistent with previously described SLs functions [31].

## 3. Discussion

The primary cilium (PC) is a key organelle for cellular signaling and tissue development, and defects in its structure are implicated in ciliopathies such as polycystic kidney disease, retinal degeneration, and cancer [5]. Identifying compounds that can restore or modulate ciliary function represents an important step toward potential therapeutic interventions. Sesquiterpene lactones (SLs) are particularly relevant in this context due to their well-documented anti-inflammatory, antitumor, and antioxidant effects [32,33], which may intersect with mechanisms of ciliary regulation. This study provides new insights into plant-derived SLs as modulators of PC structure and Hedgehog (Hh) signaling in human cells under ciliogenesis conditions (starvation) at non-cytotoxic concentrations and independently of cell cycle effects. Grosheimin, an SL from artichoke (*Cynara scolymus*), one of the oldest medicinal plants whose pharmacological mechanisms remain poorly understood, promotes ciliogenesis and preserves Hh signaling competence. In contrast, costunolide—an active SL isolated from the medicinal herb *Saussurea lappa* with demonstrated anticancer activity [32]—and its semi-synthetic derivatives α-cyclocostunolide (α-C) and β-cyclocostunolide (β-C) disrupt cilium formation and suppress Hh signaling activity. These findings extend previous observations [18] and provide functional evidence linking structural changes in the primary cilium to downstream signaling outcomes.

Grosheimin acts as a positive regulator of ciliary stability and length without inducing toxicity ([18] and this work) or interfering with Hh signaling. RNA-seq analysis suggests that this effect involves upregulation of *PLK1*, *AURKA,* and *KIF20A*, genes associated with centrosome maturation, microtubule assembly, and spindle pole organization [34,35,36,37]. Interestingly, these proteins, frequently overexpressed in tumors, play dual roles in mitosis and ciliogenesis [38,39,40]. Their upregulation by grosheimin in the absence of mitotic defects suggests reinforcement of microtubule organization, basal body stabilization, and/or ciliary trafficking under quiescent conditions, facilitating cilium assembly, elongation, and stability (Figure 5B).

In contrast, costunolide, α-C, and β-C impair ciliogenesis and reduce Hh signaling. RNA-seq of α-C-treated cells revealed broad transcriptional changes, including upregulation of DNA replication, NRF2 pathway, and cell growth genes, and downregulation of extracellular matrix components, tubulin genes (*TUBA1A*, *TUBB2A*), and vesicular trafficking elements. Repression of *PLK1* and *WNK4* further indicates interference with key ciliary regulatory nodes [38]. Previous studies have shown that other SLs, including parthenolide and costunolide, affect microtubule detyrosination and reduce stable microtubule subsets without causing global cytoskeletal collapse [41]. Our data are consistent with selective modulation of microtubule organization rather than general toxicity. Whether these transcriptional changes reflect direct effects of SLs or are secondary to altered cilium integrity remains to be clarified. Hh inhibition by costunolide derivatives likely reflects PC loss, affecting SMO trafficking and *Gli1*/*Ptch1* activation. Interestingly, increased basal *Gli1* expression in α-C-treated cells may indicate compensatory or non-canonical pathway activation. Further studies using fluorescently tagged SMO or intraflagellar transport (IFT) components will clarify how these compounds impact ciliary trafficking and signaling. Cilium assembly and disassembly are tightly regulated by mitotic kinases, vesicular transport, and tubulin modifications [38]. It is possible that SLs might act as fine-tuning agents, shifting cells toward cilia assembly or cilia disassembly states, thereby providing insight into how environmental or pharmacological factors influence ciliary function in health and disease.

The *NQO1* gene was highly overexpressed in both grosheimin- and α-C-treated cells, consistent with the antioxidant and redox-modulating activity of SLs [31]. Although several studies have suggested that redox homeostasis may intersect with ciliary regulation—potentially through oxidation-sensitive kinases such as AURKA or through modulation of vesicular trafficking [28]—our findings indicate that this activity alone does not account for the divergent effects observed on ciliary function, as the selected SLs exert opposing influences. Therefore, SL-induced alterations in redox balance might represent an additional layer of ciliary regulation.

The contrasting effects of SLs also highlight structure—activity relationships: although all four components share the α-methylene-γ-lactone moiety [19], differences in ring conformation and side chains may influence reactivity, localization, and interactions with ciliary components. Future biochemical studies are needed to identify direct molecular targets and determine whether these interactions are reversible or covalent. These opposing effects have potential therapeutic implications: grosheimin may be beneficial for ciliopathies or tumors characterized by cilium loss, whereas inhibitory costunolide derivatives could be useful against Hh-dependent cancers such as basal cell carcinoma or medulloblastoma [42]. The transcriptional profiles revealed by RNA-seq underscore the structure-dependent selectivity of SLs, offering promising chemical scaffolds for the design of cilia-targeted compounds. Importantly, all effects occurred at non-cytotoxic concentrations and independently of cell cycle alterations, confirming that ciliary modulation is a direct and pharmacologically relevant action of SLs.

Overall, plant-derived sesquiterpene lactones (SLs) can differentially modulate ciliary structure and signaling. Grosheimin and α-cyclocostunolide represent two contrasting paradigms: one enhances ciliogenesis, likely via microtubule stabilization and regulation of the expression of microtubule motor proteins essential for basal body establishment, whereas the other impairs it via repression of cytoskeletal genes, suggesting defects in axoneme formation and/or stabilization (Figure 5B). Although additional biochemical and cellular studies are needed to validate and further elucidate their precise mechanisms, these findings provide a framework for exploring SLs as potential modulators of cilia-related pathways for the structure-guided design of cilia-targeted therapeutics, and they contribute to a deeper understanding of cilia biology.

## 4. Materials and Methods

### 4.1. Extraction, Isolation, and Semi-Synthesis of Sesquiterpene Lactones Compounds

Product purification and isolation were performed as previously described [18]. Briefly, costunolide was isolated from a 50 g root extract of *Saussurea lappa* (purchased from Pierre Chauvet S. A, Seillans, France), while grosheimin was obtained from a 12.5 g leaf extract of *Cynara scolymus* (12.5 g). Semi-synthesis of α-cyclocostunolide and β-cyclocostunolide was carried out by stirring isolated costunolide in CH_2_CL_2_ and p-TsOH to induce cyclization. All products were validated by spectroscopic analysis.

### 4.2. Cell Culture Conditions and Biological Assays

The hTERT-immortalized retinal pigment epithelial cell line (hTERT RPE-1) was provided by Dr. Fernando Balestra at Cabimer, Seville, Spain. Human primary fibroblasts (RHDFs) were provided by Dr. Lechuga ( INiBICA, Cadiz), following all ethical committee approvals and using the extraction protocol available at https://dx.doi.org/10.17504/protocols.io.81wgbrrjylpk/v2 (accessed on 15 March 2025). RPE and fibroblasts were grown at 37 °C in 5% CO_2_ in DMEM/F-12 and DMEM-Low glucose, respectively. Media were supplemented with L-glutamine, sodium pyruvate, 10% FBS (Fetal Bovine Serum) (Gibco, Thermo Fisher Scientific, Waltham, MA, USA), 100 U/mL penicillin, 100 µg/mL streptomycin, and 3 µg/mL ciprofloxacin (NORMON laboratories, Madrid, Spain).

For cilia assays, RHDF cells were seeded at a density of 15,000–20,000 cells/well in 12-well plates, and experiments were performed after 72 h. RPE cells were seeded at 70,000 cells/well in 12-well plates, and experiments were performed after 24 h. Cilia were induced by incubating cells for 24 h in media supplemented with 0.5% FBS (starvation). For experiments aimed at seeing the effect of test compounds on cilia formation, SLs were added simultaneously with the 0.5% FBS media at a final concentration of 10 µM. For experiments assessing the effect of test compounds on established cilia, after 24 h in 0.5% FBS media, test compounds were added for an additional 24 h in 0.5% FBS media at a final concentration of 10 µM. DMSO was used as the control condition, as test compounds were diluted in DMSO stock solution.

### 4.3. Hedgehog Pathway Activation

Confluent RDHF fibroblasts were seeded into 6-well plates and starved for 24 h, then treated with (1) Hedgehog pathway activators: recombinant human Sonic Hedgehog (SHH 8908-SH/CF, R&D Systems, Minneapolis, MN, USA) at a final concentration 61.6 ng/mL and Smoothened Agonist (SAG 566660, Merck KGaA, Darmstadt, Germany) at 750 nM, and (2) 10 μM of product or control DMSO in serum starved-media for 24 h. RNA was collected 24 h post-treatment using gTPzol (gTPBio).

### 4.4. Immunofluorescence (IF)

Cells were seeded onto coverslips in 12-well plates, and cilia experiments were performed as described above. Coverslips were fixed with −20 °C cold methanol for 10 min, washed with 1X PBS, and blocked for 1 h at room temperature (RT) in IF blocking buffer (5% [*wt*/*vol*] BSA, 0.05% [*vol*/*vol*] Tween-20 in 1X PBS). Cells were incubated with primary antibodies diluted in blocking buffer overnight at 4 °C, washed three times for 5 min in 1X PBS containing 0.05% *(vol*/*vol*) Tween-20, and incubated with secondary fluorescent antibodies for 1 h at RT. Coverslips were washed three times, incubated with DAPI (5 μg/mL, Sigma-Aldrich, St. Louis, MO, USA) for 5 min, washed, and mounted in glycerol-based 2.5% [*wt*/*vol*] PVA-DABCO mounting medium for imaging. Primary antibodies: γ-tubulin (1:1000, Sigma T3559) and acetylated-tubulin (1:2000, Sigma T7451). Secondary antibodies: Alexa 488 conjugated anti-mouse A32723 (1:500, Invitrogen, Life Technologies, Eugene, OR, USA) and Alexa 568-conjugated anti-rabbit (1:500, Invitrogen A11011).

### 4.5. Microscopy

Images were collected at room temperature using a Zeiss LSM 900 inverted confocal microscope (Carl Zeiss, Jena, Germany) with a 40X 1.3 NA oil-immersion objective and Zeiss ZEN software 3.2. Images were acquired as 0.5-µm z sections. Maximum intensity projections are shown in figures. Fluorophores imaged are those conjugated to the secondary antibodies listed above. Images were z-stacked and converted to JPG using Fiji 1.53c, and analyzed for cilia proportion and length both manually (Fiji 1.53c) [43] and semi-automatically using ACDC software_v0.93 [44] in MATLAB R2016b. A 4× magnification of images was included for better visualization of cilia and/or centrosomes.

### 4.6. RNA Preparation and Sequencing

RPE cells from 2 × 12-well plates for each condition were collected using a scraper in RLT buffer (RNeasy Mini kit 74104, QIAGEN, Hilden, Germany), vortexed, column-cleaned, DNase-treated (RNase free DNase Set, Qiagen 79254), and column-cleaned again according to the manufacturer’s instructions. RNA quality was assessed, and 150 ng was used to prepare libraries following the Illumina Stranded mRNA Prep Ligation protocol (Illumina, San Diego, CA, USA). Libraries were quality-checked using TapeStation DNA High Sensitivity D1000 assay and quantified using Qubit™ DNA HS Assay. Library sizes were approximately 270–290 bp. Sequencing was performed on a NextSeq 500 (MID-150 cycles, 2 × 75 bp, *paired*-*end*). BaseSpace Seq Hub (Illumina, San Diego, CA, USA) indicated that sequencing quality was high (>93% ≥ Q30).

### 4.7. Quantitative PCR (qPCR) Analysis

Human fibroblasts were cultured under starvation and Hedhehog pathway activation as described above. Total RNA was extracted using gTPzol and DNase-treated. cDNA was synthesized using iScript gDNA Clear cDNA Synthesis Kit (BioRad, Hercules, CA, USA) according to the manufacturer’s instructions. PCR products were amplified using following primer sets: hGli1 Fw: 5′-AATGCTGCCATGGATGCTAGA-3′, hGli1 Rv: 5′-GAGTATCAGTAGGTGGGAAGTCCATAT-3′, hPtcch1 Fw: 5′-TCTCCAATCTTCTGGCGAGT-3′, hPtch1 Rv: 5′-TGGGATTAAAAGCAGCGAAC-3′, hGAPDH Fw: 5′-TCAAGGCTGAGAACGGGAAG-3′, hGAPDH Rv: 5′-CGCCCCACTTGATTTTGGAG-3′, hTBP Fw: 5′-GTGACCCAGCATCACTGTTTC-3′, and hTBP Rv: 5′-AGAGCATCTCCAGCACACTC-3′. *GADPH* and *TBP* were used as housekeeping controls. qPCR reactions were performed using iTaq SYBR Green Supermix (BioRad, Hercules, CA, USA) in 10 mL volumes on a QuantStudio^TM^ 12K Flex Real-Time PCR System (ThermoFisher, Waltham, MA, USA). Cycling conditions: 50 °C for 2 min and 95° for 10 min (hold), 40 cycles of 95 °C for 15 s and 60 °C for 1 min (PCR), followed by a melt curve. Relative gene expression was calculated using the ΔΔCt method.

### 4.8. RNA-Seq Analysis

FASTQ files were first examined using FASTQC from https://www.bioinformatics.babraham.ac.uk/projects/fastqc/ (accessed on 5 May 2025). Reads were aligned to the human reference genome (GRCh38/hg38) using STAR [45], and first-strand paired-end fragments were quantified per gene using featureCounts. TPMs were calculated, and differential expression analysis was performed with DESeq2 [46] in R, comparing grosheimin vs. control (DMSO) and α-cyclocostunolide vs. control (DMSO). Significant genes (adjusted *p* < 0.05) were used for gene list creation for upregulated and downregulated genes. Over-representation analysis was performed with ClusterProfiler to identify enriched biological ontologies. Primary cilium-related genes were referenced from Reiter et al., 2017 [29].

### 4.9. Statistical Analysis

All experiments were performed at least in triplicate (when *n* > 3, indicated in figure legend). Approximately 100 cells per condition were analyzed for immunofluorescence. Data are expressed as a ratio of mean values relative to DMSO control (0.1%) ± SEM. Statistical analysis was performed using GraphPad Prism 10.5.0 (Boston, MA, USA). Mann–Whitney tests were used to assess differences between conditions. For qPCR experiments, *n* = 6 experiments. *p*-values for each experiment are indicated in the figure legends.

## 5. Conclusions

Our study demonstrates that plant-derived sesquiterpene lactones differentially modulate primary cilium structure and Hedgehog (Hh) signaling in vitro. Grosheimin promotes ciliogenesis and preserves signaling competence, whereas costunolide and its derivatives disrupt cilia formation and inhibit pathway activation. RNA-seq analysis suggests that grosheimin enhances ciliogenesis through stabilization of the microtubule-organizing ceknter (MTOC), while α-cyclocostunolide impairs it by repressing cytoskeletal gene expression. These effects occur at non-toxic concentrations and independently of cell cycle alterations, highlighting specific structure–activity relationships. Together, our findings uncover novel mechanisms by which natural compounds can fine-tune ciliary biology and signaling, providing a foundation for future exploration of sesquiterpene lactones as potential therapeutic modulators in ciliopathies and cilia-related cancers.

## Data Availability

Data supporting RNA-seq analysis can be found in ArrayExpress, accession number: E-MTAB-15939 and the codes used have been added to the GitHub platform: https://github.com/SantpereLab/TestProducts.git (accessed on 5 September 2025).

## Abbreviations

The following abbreviations are used in this manuscript:

PC	Primary Cilium
SL	Sesquiterpene Lactones
Hh	Hedgehog
C	Costunolide
α-C	α-Cyclocostunolide
β-C	β-Cyclocostunolide
SMO	Smoothened
IFT	Intraflagellar Transport
RPE	Retinal Pigment Epithelial
RHDF	Human primary fibroblasts
